# Expression and function of four AAV-based constructs for dystrophin restoration in the *mdx* mouse model of Duchenne muscular dystrophy

**DOI:** 10.1242/bio.059797

**Published:** 2023-09-18

**Authors:** Rachael A. Potter, Danielle A. Griffin, Kristin N. Heller, Jerry R. Mendell, Louise R. Rodino-Klapac

**Affiliations:** ^1^ Center for Gene Therapy, The Research Institute at Nationwide Children's Hospital, Columbus, OH 43205, USA; ^2^ Sarepta Therapeutics, Inc., Cambridge, MA 02142, USA; ^3^ Department of Pediatrics and Neurology, The Ohio State University, Columbus, OH 43210, USA

**Keywords:** Adeno-associated virus, Duchenne muscular dystrophy, Dystrophin, Gene therapy, *Mdx* mouse model, Micro-dystrophin

## Abstract

Robust expression of shortened, functional dystrophin provided impetus to develop adeno-associated virus (AAV)–based constructs for clinical application. Because several cassettes are being tested in clinical trials, this study compared the efficacies of four shortened dystrophin-promoter combinations with implications for outcomes in clinical trials: MHCK7 or MCK promoter with a shortened dystrophin transgene containing the N-terminus and spectrin repeats R1, R2, R3 and R24 (rAAVrh74.MHCK7.micro-dystrophin and rAAVrh74.MCK.micro-dystrophin, respectively); shortened dystrophin construct containing the neuronal nitric oxide (nNOS) binding site (rAAVrh74.MHCK7.DV.mini-dystrophin); and shortened dystrophin containing the C-terminus (rAAVrh74.MHCK7.micro-dystrophin.Cterm). Functional and histological benefit were examined at 4 weeks following intramuscular delivery in *mdx* mice. rAAVrh74.MHCK7.micro-dystrophin provided the most robust transgene expression and significantly increased specific force output in the tibialis anterior muscle. Muscle environment was normalized (i.e. reductions in central nucleation), indicating functional and histological advantages of rAAVrh74.MHCK7.micro-dystrophin. Thus, promoter choice and transgene design are critical for optimal dystrophin expression/distribution for maximal functional improvement.

## Teaser

**Summary:** Comparing four shortened dystrophin combinations in an rAAVrh74 platform showed that rAAVrh74.MHCK7.micro-dystrophin produced the greatest functional improvement in *mdx* mice, highlighting the importance of promoter/transgene selection in gene transfer therapies.

## INTRODUCTION

Duchenne muscular dystrophy (DMD) is a degenerative, neuromuscular disease caused by mutations that disrupt the open reading frame of the X-linked dystrophin gene and occurs in approximately one per 3500 to 5000 males worldwide (Bushby et al., 2010; Emery, 1991; Mendell et al., 2012; Mendell and Lloyd-Puryear, 2013). The lack of a functional dystrophin protein leads to destabilization of cardiac and skeletal muscle fibers, turnover of muscle tissue, satellite cell depletion, and replacement of fibers by fat and fibrosis (Koenig et al., 1987; Webster and Blau, 1990). Ultimately, patients with DMD experience a progressive loss of muscle strength and ambulation, leading to respiratory and cardiac failure, and eventually death (Brooke et al., 1983; Hoffman et al., 1987).

Treatment with corticosteroids delays DMD disease progression, but the underlying cause is not addressed, and long-term use is associated with serious adverse effects (Birnkrant et al., 2018; Liu et al., 2013). Eteplirsen was the first disease-modifying treatment approved for patients with DMD amenable to exon 51 skipping therapy and has been shown to produce functional dystrophin protein and to slow the decline in ambulatory and pulmonary function with long-term use (Alfano et al., 2019; Cirak et al., 2011; Khan et al., 2019; Mendell et al., 2016). More recently, other disease-modifying treatment options have been approved for the treatment of DMD amenable to exon 53 and 45 skipping (Clemens et al., 2020, 2022; Frank et al., 2020; Iannaccone et al., 2021; Scaglioni et al., 2021).

Systemic adeno-associated virus (AAV) gene transfer therapy is also being evaluated in clinical trials as a one-time intervention to restore the production of a functional dystrophin protein in patients with DMD. Gene transfer of the full-length dystrophin gene is not feasible because of its large size relative to the packaging capacity of AAV-derived vectors (∼5 kb). Early clinical data showed that large deletions in the dystrophin gene that do not disrupt the open reading frame in patients with the milder, Becker muscular dystrophy result in partially normal muscle structure and function (England et al., 1990). These data suggest that delivery of shortened but functional forms of dystrophin that include key structural and functional domains may be a viable strategy that could overcome the constraints of the AAV packaging capacity. Proof of principle has been established in preclinical studies showing that shortened forms of dystrophin (micro-dystrophin) have the potential to ameliorate muscle pathology and cardiomyopathy when delivered in multiple DMD animal models (Gregorevic et al., 2008; Koo et al., 2011; Le Guiner et al., 2017; Liu et al., 2005; Shin et al., 2013; Yue et al., 2003).

Questions remain regarding which components (i.e., vector, promoter and transgene) are best suited to impact the safety and efficacy of treatment. The approach has been used previously by Harper et al. (2002), setting the stage for small dystrophin entry into clinical trials. While their approach clearly has merit, comparing the efficacy of shortened (four or five spectrin repeats) combined with different promoters has contemporary relevance to current approaches undergoing evaluation in clinical trials (Elangkovan and Dickson, 2021; Mendell et al., 2020).

**Fig. 1.**
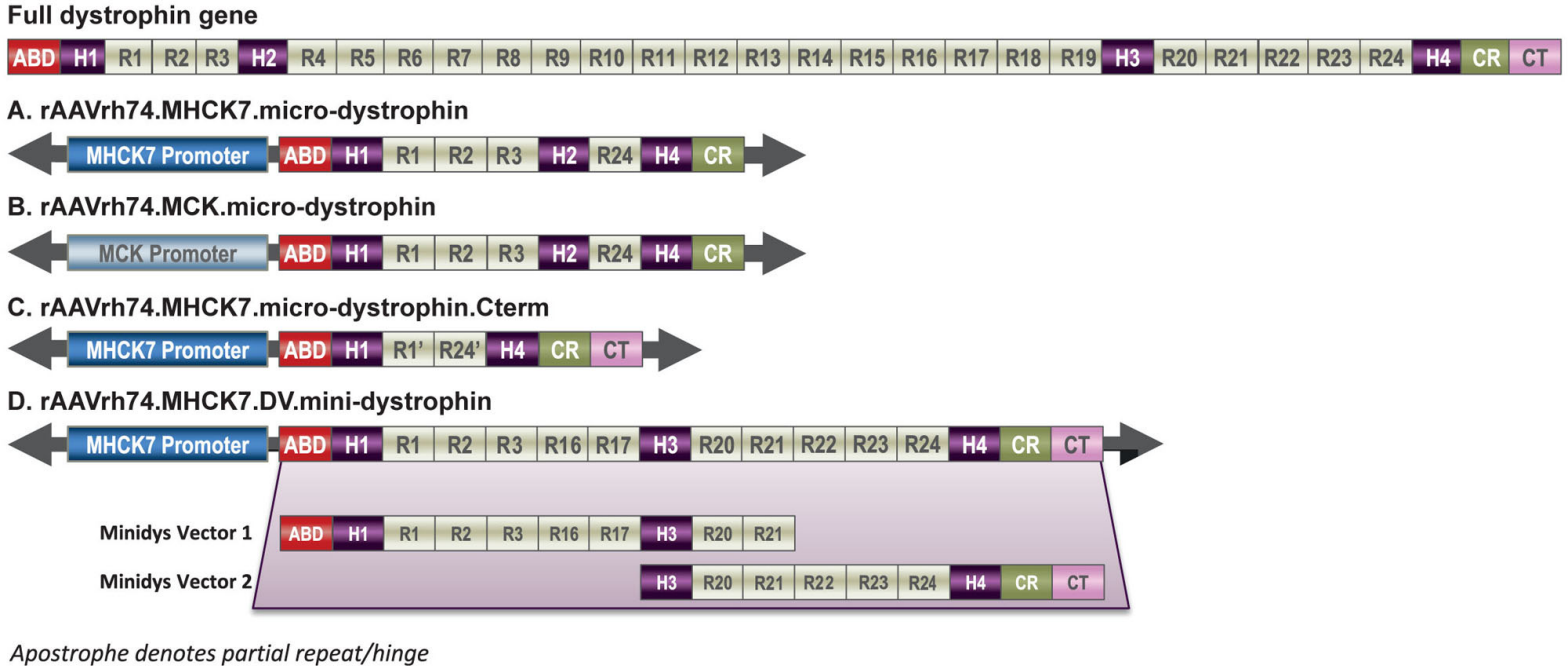
**Full-length dystrophin and construct schematics.** Spectrin-like repeats 1-3 (R1-3) have been shown to be essential for maximal protection against eccentric force loss. R16/17 contains an nNOS binding domain (Hakim et al., 2017). Hinge domains (H1-4) are important for conferring flexibility, but no differences in restoration of muscle functional capacity have been found between H2 and H3 (Banks et al., 2010). ‘ indicates partial repeat. The MCK promoter directs tissue-specific expression in both skeletal and cardiac muscle while the MHCK7 promoter drives robust expression selectively in skeletal and cardiac muscle through the addition of α-MHC that enhances expression in cardiac muscle (Salva et al., 2007; Rodino-Klapac et al., 2007, 2013). ABD, actin binding domain; CR, cysteine-rich domain; CT, C-terminus; R1-24, spectrin-like repeats 1-24.

Identification of the optimal shortened dystrophin construct that most normalizes muscle function is a key translation question. Another key goal of AAV-mediated gene therapy for DMD is to achieve dystrophin transgene expression in both skeletal and cardiac muscle, especially as deterioration of the latter greatly increases mortality risk (Bushby et al., 2010). The purpose of this study was to evaluate various shortened dystrophin constructs in terms of their ability to restore dystrophin function and the optimal promoter regions to drive robust expression of the shortened dystrophin transgene in skeletal muscle. The dystrophin protein has an N-terminal actin binding domain (ABD), 24 spectrin-like repeats (R), a cysteine-rich domain (CRD), and a carboxy terminus (CT) interspersed with four critical hinge regions (H1-H4). In the present study, we report the expression and functional characteristics of four different shortened dystrophin vector constructs using a recombinant adeno-associated virus (rAAV) delivery system in a dystrophin-null (*mdx*) mouse model of DMD (Fig. 1). The shortened dystrophin contains the functional elements that have been tested in preclinical studies (Gao and Mcnally, 2015) or observed in the clinic (England et al., 1990): the ABD and CRD; spectrin-like repeats R1-3 with membrane-binding properties (Cooper-Olson et al., 2021; Zhao et al., 2016) that have shown to protect muscle tissue from damage by modulating radial force transmission and mechanical vulnerability in preclinical studies (Nelson et al., 2018); spectrin-like repeat R24, necessary for microtubule organization (Nelson et al., 2018); and H1, H2 and H4, short proline-rich spacers that provide elasticity to the protein. In this micro-dystrophin construct, H2 is positioned just after R3 as in the naturally occurring dystrophin, resulting in a construct with fewer novel junctions (between H2 and R24) to minimize alterations in the final protein structure. We also tested the micro-dystrophin.C-term construct that has shown functionality in a previous study (Jørgensen et al., 2009) and includes the ADB; H1 and H4; two spectrin-like repeats and the CR plus the CT domain. Finally, we tested a larger mini-dystrophin transgene that included all previous elements mentioned (ABD, H1, H3, H4, R1-3, R24, CR and CT) as well as spectrin-like repeats R16-17 and R20-23. Due to the large size of this construct, this was designed in a dual vector delivery system, allowing for a comparison between single versus dual vector systems. In addition, we compared two different muscle-specific promoters that have previously been used, muscle creatine kinase (MCK) and an enhanced cardiac and skeletal muscle promoter MHCK7, for their ability to maximize gene expression in the relevant tissues and mitigate any off-target effects (Rodino-Klapac et al., 2007; Salva et al., 2007). The MHCK7 promoter includes an embryonic α-myosin heavy chain (α-MHC) enhancer that leads to higher levels of expression in cardiomyocytes and increased dystrophin expression in skeletal muscle compared with the MCK promoter (Rodino-Klapac et al., 2010, 2013; Salva et al., 2007).

## RESULTS

### Shortened dystrophin expression

**Fig. 2.**
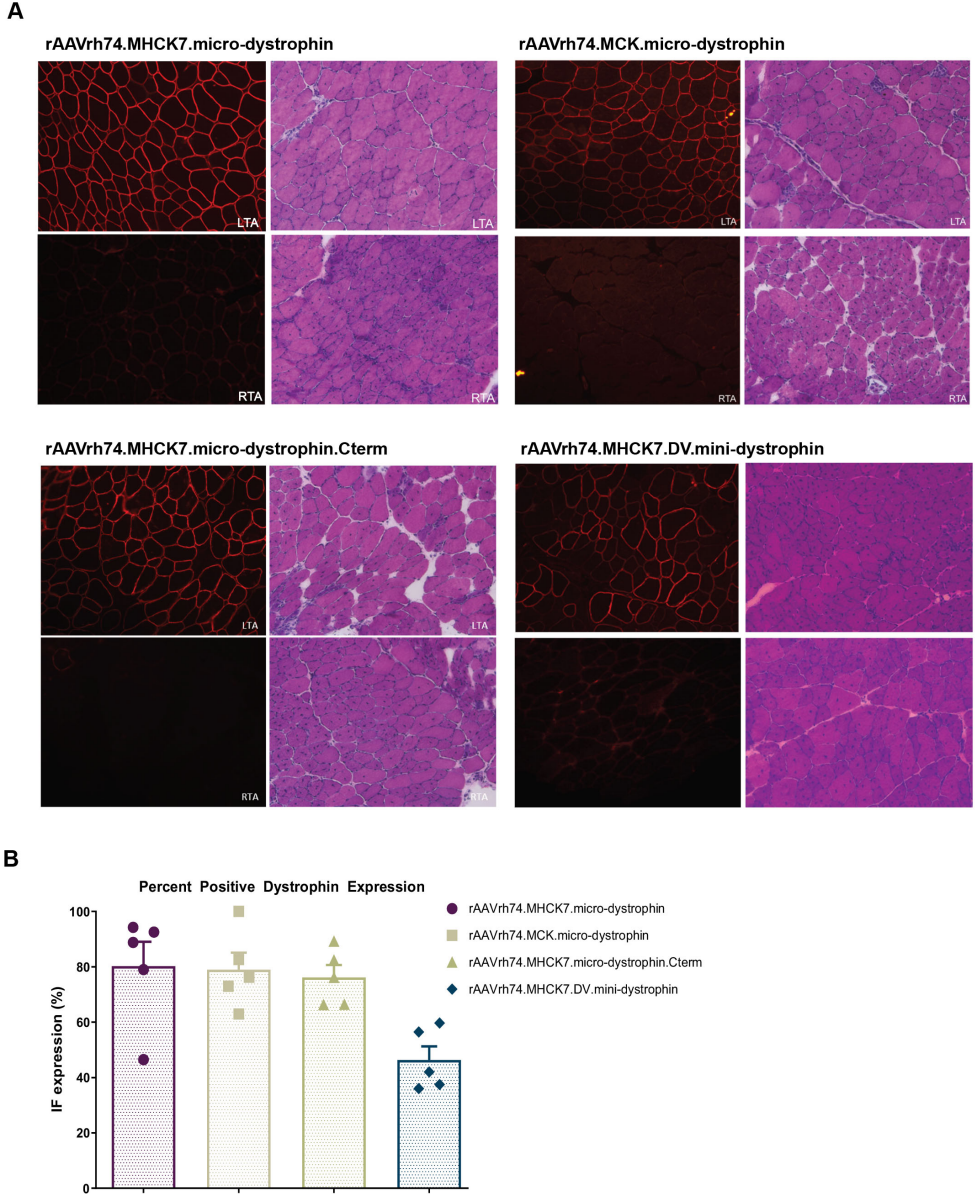
**Dystrophin expression levels and improvement in muscle morphology depends on promoter and transgene.** Mice received vector constructs intramuscularly in the left tibialis anterior (LTA). The untreated right tibialis anterior (RTA) is the contralateral (untreated) limb. (A) Representative immunofluorescent staining images for shortened dystrophin 4 weeks post-injection. 20× images are shown. (B) Quantification of dystrophin fiber expression assessed by immunofluorescence (*n*=5 for each cohort). Data are expressed as individual values and the mean±s.e.m.

Four weeks after transgene delivery, shorted dystrophin protein expression was quantified using immunofluorescence staining for dystrophin-positive fibers. Fibers positive for dystrophin expression were higher in the left tibialis anterior (LTA) treated with rAAVrh74.MHCK7.micro-dystrophin, rAAVrh74.MCK.micro-dystrophin, and rAAVrh74.MHCK7.micro-dystrophin.Cterm constructs compared to those treated with rAAVrh74.MHCK7.DV.mini-dystrophin (Fig. 2A,B). Mean (s.e.m.) percentage positive dystrophin expression in the LTA of treated mice was 80±8.8%, 78±6.1%, and 76±4.5% (*n*=5 for each cohort), respectively, in mice treated with the rAAVrh74.MHCK7.micro-dystrophin, rAAVrh74.MCK.micro-dystrophin, and rAAVrh74.MHCK7.micro-dystrophin.Cterm constructs. In contrast, dystrophin expression was appreciably lower (50±4.9%) in mice treated with the rAAVrh74.MHCK7.DV.mini-dystrophin construct.

Dystrophin intensity was quantified using BIOQUANT and was based on four images (20x) of the injected TA. The intensity at the membrane for each construct was as follows: rAAVrh74.MHCK7.micro-dystrophin [38,763 (SD: 21,731)], rAAVrh74.MCK.micro-dystrophin [26,122 (SD: 11,824)], rAAVrh74.MHCK7.micro-dystrophin.Cterm [46,257 (SD: 15,424)], and rAAVrh74.MHCK7.DV.mini-dystrophin [7742 (SD: 10,393)]. No statistically significant differences in transduction efficiency [vector genome (vg) copies per nucleus] were demonstrated across the constructs (Table 1).

**Table 1.**
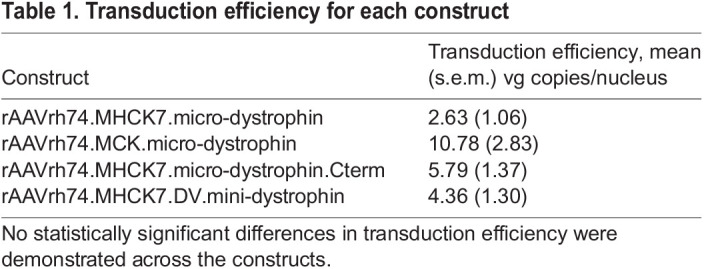
Transduction efficiency for each construct

### Western blotting

**Fig. 3.**
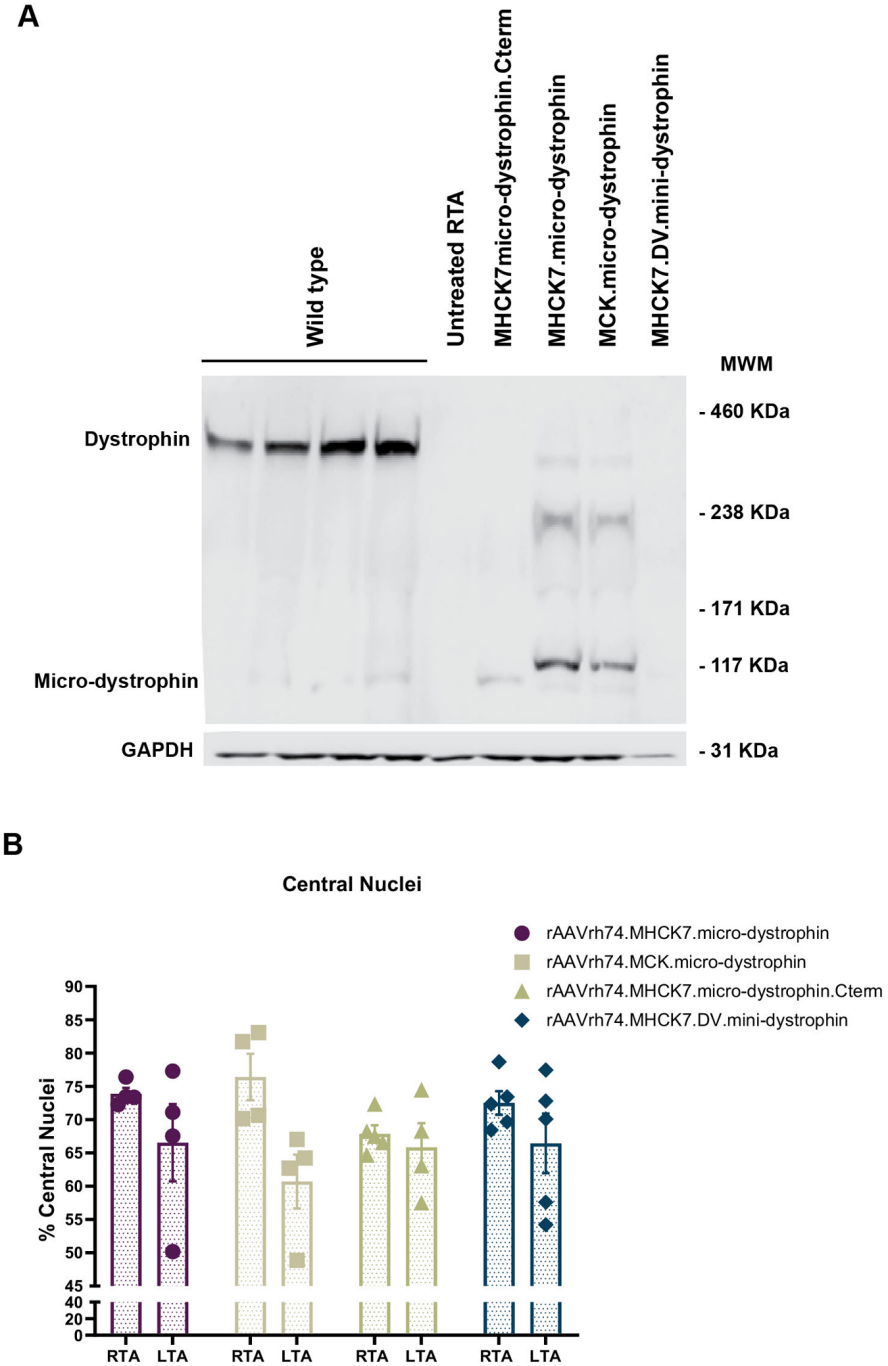
**Dystrophin protein expression following intramuscular delivery of gene therapy constructs.** (A) Representative Western blot for shortened dystrophin protein expression in untreated TA (far left lane) of *mdx* mice compared with TA treated with the indicated constructs. (B) Improvement in dystrophic pathology (i.e. reduction in central nucleation) was observed in treated TA (LTA) after treatment with the constructs with the micro-dystrophin transgene (*n*=4 for each cohort). Data are expressed as individual values and the mean±s.e.m.

Shortened dystrophin protein expression was absent in untreated mice as expected and detectable in mice treated with rAAVrh74.MHCK7.micro-dystrophin and rAAVrh74.MCK.micro-dystrophin (Fig. 3A). Dystrophin protein expression was higher in mice receiving the rAAVrh74.MHCK7.micro-dystrophin construct than in those animals receiving the rAAVrh74.MCK.micro-dystrophin construct (% wildtype dystrophin expression, 8.59 versus 2.69, respectively). Relative to these two shortened dystrophin constructs, expression was considerably lower in mice treated with rAAVrh74.MHCK7.micro-dystrophin.Cterm (% wildtype dystrophin expression, 0.1) and was below the level of detection in mice treated with the rAAVrh74.MHCK7.DV.mini-dystrophin.

### Histopathology

Reduction in central nucleation, consistent with a normalization in the muscle environment and improvement in dystrophic pathology by reducing degeneration/regeneration, was seen in the LTA compared with the contralateral untreated control [right tibialis anterior (RTA)] of mice receiving the rAAVrh74.MHCK7.micro-dystrophin (10% reduction) and rAAVrh74.MCK.micro-dystrophin (16% reduction) constructs although the reductions were not statistically significant (Fig. 3B). The percentage of central nucleation was only reduced by 3% in mice receiving rAAVrh74.MHCK7.micro-dystrophin.Cterm or 6% in mice receiving rAAVrh74.MHCK7.DV.mini-dystrophin.

### Muscle function

**Fig. 4.**
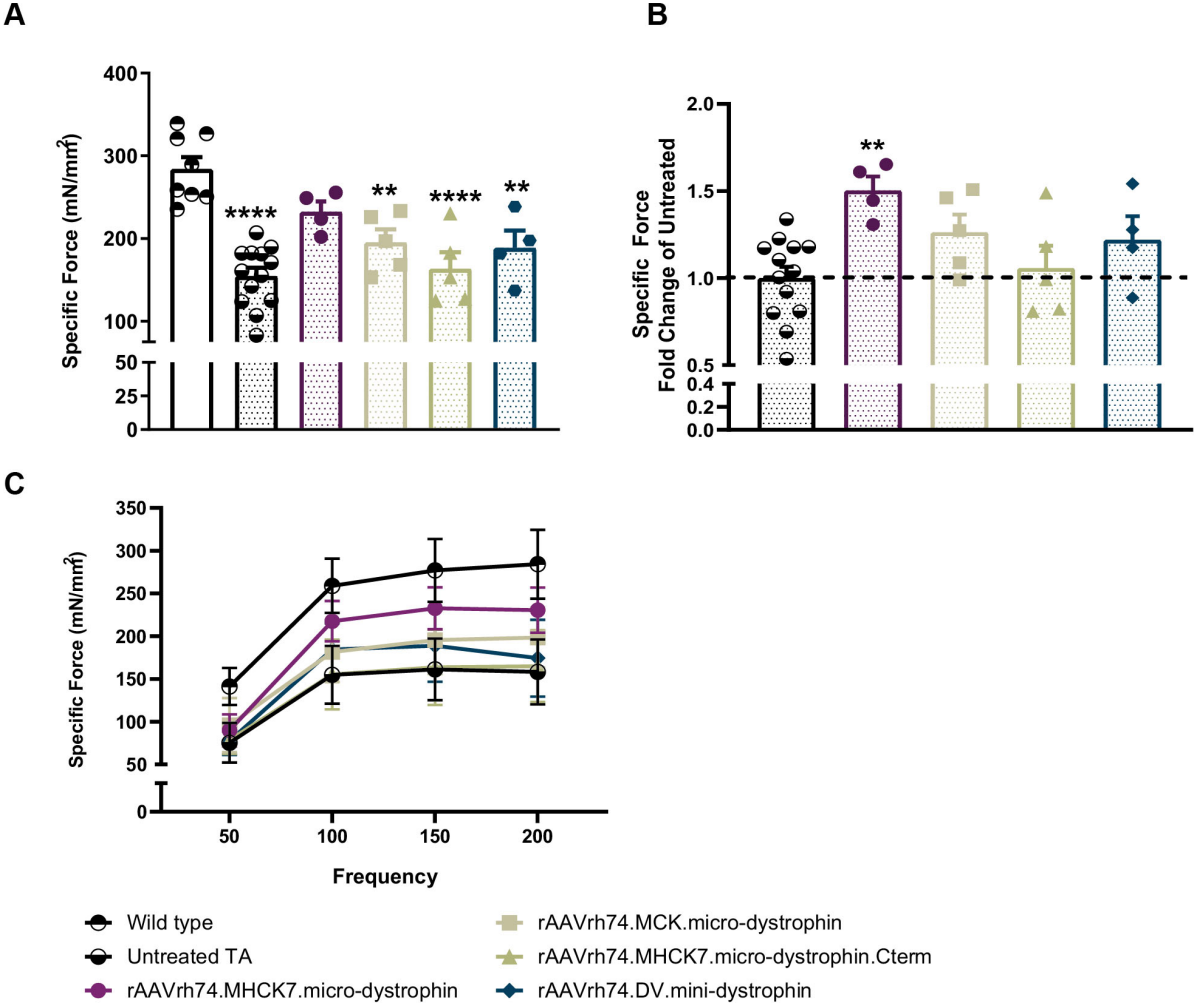
**rAAVrh74.** MHCK7.micro-dystrophin produces the greatest functional improvement in muscle contractile performance. A and B, 4 weeks after treatment, left and right tibialis anterior muscles were harvested to measure specific force, normalized to tibialis anterior weight (A) specific force, (B) fold change in specific force, and (C) specific force frequency. Data are expressed as individual values and the mean±s.e.m. (untreated *n*=13; wild type *n*=8; treated *n*=5). Statistical analysis was performed using one-way ANOVA with multiple comparisons between groups with Turkey's post-hoc analysis. Panel A ****P<0.0001; **P<0.004 denotes the statistical difference between treated and wild-type TA groups. Panel B **P<0.0086 denotes the statistical difference between the rAAVrh74.MHCK7.micro-dystrophin and untreated TA groups.

There was a significant reduction in specific force output of the TA muscle from untreated control *mdx* mice compared with wild-type C57BL/6 mice (154.7±10.09 mN/mm^2^ vs 284.2±14.23 mN/mm^2^; *P*<0.0001) (Fig. 4A). Four weeks after transgene delivery, specific force output was increased in all groups treated with shorted dystrophin constructs. The largest increase was seen after delivery of rAAVrh74.MHCK7.micro-dystrophin compared with untreated *mdx* mice (232.7±12.28 mN/mm^2^ versus 154.7±10.09 mN/mm^2^; *P*<0.0117). The observed specific force for this group was comparable to wild-type C57BL/6 mice without significant difference (*P*=0.251; Fig. 4A). No other construct achieved specific force output in the normal range.

Consistent with the results observed with dystrophin expression with respect to rAAVrh74.MHCK7.micro-dystrophin, the increase in specific force output of the TA muscle of mice receiving the rAAVrh74.MHCK7.micro-dystrophin was 15% maximal increase compared with the untreated control group (*P*=0.0086) (Fig. 4B). In contrast, the specific force output in the TA muscle from *mdx* mice receiving the other constructs was not significantly different from that observed in untreated *mdx* mice (Fig. 4B). In addition, the specific force frequency was greatest with the rAAVrh74.MHCK7.micro-dystrophin construct (Fig. 4C).

## DISCUSSION

In the search for ideal candidates for gene therapy treatments, it is imperative to evaluate safety, transduction, expression, localization, cellular impact and functional outcomes of the chosen candidate and compare it with similar other promoter-transgene combinations. In this preclinical study, the combination of the MHCK7 promoter and shortened dystrophin transgene generated optimal dystrophin transgene expression and increased functional benefit in *mdx* mice compared with the other constructs analyzed.

All constructs were designed in an rAAVrh74 vector platform to maximize delivery to muscle tissue. rAAVrh74, a serotype isolated from rhesus monkeys, displays high affinity for skeletal and cardiac muscle tissue (Chicoine et al., 2014; Pozsgai et al., 2016, 2017) and has been shown to be well tolerated in several clinical trials (Mendell et al., 2010, 2019, 2020). Transgene expression was analyzed measuring protein production through immunofluorescence microscopy in muscle cells. Results demonstrated a high percentage of dystrophin-positive fibers and protein localization at the sarcolemma when mice were treated with the rAAVrh4.micro-dystrophin vector with either the MHCK7 or MCK promoter (80% and 78%, respectively) as well as the construct inclusive of the C-terminus (76%). However, quantification of protein expression showed significant differences that were dependent on the promoter. Quantification of the immunofluorescence intensity for the two shortened dystrophin constructs showed an increase of 55.6% for rAAVrh74.MHCK7.micro-dystrophin compared to rAAVrh74.MCK.micro-dystrophin. In addition, qualitative Western blot analysis provided similar results, showing the most intense dystrophin protein band for the rAAVrh74.MHCK7.micro-dystrophin construct (Fig. 3A). Much lower intensity was observed for the rAAVrh74.MHCK7.micro-dystrophin.Cterm construct, suggesting that the transgene design could affect protein stability. We also compared single and dual vector systems. We could not observe a dystrophin protein band for the mini-dystrophin construct, which could be related to lower transduction efficiency of the dual vector strategy when compared with a single vector (McClements and Maclaren, 2017; Tornabene and Trapani, 2020).

Protein expression was associated with functional improvements in specific force output in the TA muscle that was most significant with the rAAVrh74.MHCK7.micro-dystrophin construct. In fact, specific force output was similar in TA muscles from *mdx* mice receiving the rAAVrh74.MHCK7.micro-dystrophin construct to that observed in the TA muscle of wild-type mice, suggesting that this construct almost completely reversed the functional impairment seen in dystrophin-null mice. The data illustrate the importance of choosing the optimal promoter to drive transgene expression, which is required for functional improvement. The importance of promoter selection in driving shortened dystrophin expression has previously been described by Sarcar and colleagues, where the specific muscle promoters analyzed increased transgene expression in skeletal muscles compared with promoters frequently used for muscle gene therapy (CMV and SPc5-12 synthetic promoter) (Sarcar et al., 2019).

The importance of the transgene optimization was also analyzed with the rAAVrh74.MHCK7.micro-dystrophin.Cterm construct. Protein expression for the C-terminal transgene was similar to the other constructs by percentage of positive fibers. However, qualitative Western blot analysis showed much lower expression for the shorter rAAVrh74.MHCK7.micro-dystrophin.Cterm construct, suggesting that the design of the transgene could affect protein stability. We hypothesize that the absence of R1-R3 may at least partially explain the lack of improvement in muscle function seen in animals receiving this construct. Hinge domains are important for conferring flexibility (Cooper-Olson et al., 2021; Koenig and Kunkel, 1990), but no differences in restoration of muscle functional capacity have been found between H2 and H3 (Banks et al., 2010). One study suggested inclusion of H2 versus H3 may influence the functional capacity of shortened dystrophin by altering the structure of the tendinous and neuromuscular junctions and reducing myofiber size (Banks et al., 2010). In the present study and others (Chicoine et al., 2014; Potter et al., 2021; Rodino-Klapac et al., 2010) as well as clinical study results (Mendell et al., 2020), the absence of the H3 domain did not adversely impact dystrophin expression or muscle functionality in mice receiving the rAAVrh74.MHCK7.micro-dystrophin construct. This is consistent with a previous study in which the function of various shortened dystrophin genes was compared, showing that the absence of H3 did not impact the functionality of some constructs analyzed (Ramos et al., 2019).

Inclusion of other spectrin-like repeat modules (R16-17 and R20-R23) and the C-terminus domain in the rAAVrh74.MHCK7.DV.mini-dystrophin construct, the largest version of the dystrophin transgene, did not appear to remarkably improve functional outcomes. This is likely to be associated with a substantially lower dystrophin expression observed compared with the other constructs.

The rAAVrh74.MHCK7.micro-dystrophin construct has also been evaluated in a dose ranging study in *mdx* mice using similar functional and histological evaluations to those employed in the current study (Potter et al., 2021). *Mdx* mice received intravenous injections of rAAVrh74.MHCK7.micro-dystrophin at low (2×10^12^ vg total; 8×10^13^ vg/kg), intermediate (6×10^12^ vg total; 2×10^14^ vg/kg), and high doses (1.2×10^13^ vg total; 6×10^14^ vg/kg). At 3 months post treatment, specific force was increased in the diaphragm and TA muscles from mice receiving the intermediate and high doses, reaching force output levels similar to those achieved in muscle tissue from wild-type mice. These functional improvements were accompanied by a reduction in fibrosis and centralized nuclei, and a normalization of myofiber size in the intermediate- and high-dose groups (Potter et al., 2021). Furthermore, results from a clinical trial initiated to test the safety and tolerability of this same construct are promising (Mendell et al., 2020).

The present study is limited by the route of AAV administration used given that intravenous delivery of AAV gene therapies in the DMD population is necessary. Intramuscular delivery was selected to directly compare cassette design using a robust delivery technique with comparable levels of vector genome biodistribution of different constructs (Rodino-Klapac et al., 2010; Sondergaard et al., 2015). Direct intramuscular delivery enabled robust transduction with no statistically significant differences in level of transduction across constructs, expression and direct muscle force evaluation. Furthermore, the use of intramuscular delivery as proof of concept in AAV directed gene therapy has been previously published prior to expanding into systemic dose escalation studies (Rodino-Klapac et al., 2010). Additionally, only one muscle was injected in the present study; therefore, there is no guarantee that the observations reported in the present study would be similar in other muscles. Moreover, the rAAVrh74.MHCK7.DV.mini-dystrophin construct may not represent an adequate comparator given the confounding challenge of the dual vector constructs. Finally, the present study did not evaluate the impact of an nNOS domain in a single vector as it was completed prior to the initiation of the clinical trials.

In conclusion, administration of the rAAVrh74.MHCK7.micro-dystrophin construct to the TA muscle of *mdx* mice resulted in histological and functional improvements in dystrophic pathology. The shortened dystrophin transgene sequence (ΔR4-23/ΔCT) driven by an MHCK7 promoter produced the most advantageous outcome measures of the four analyzed constructs in the *mdx* mouse model. The results of this study highlight the importance of selecting an optimal combination of vector, promoter, and transgene sequence design as each of these components play a key role in the optimization of dystrophin expression, correct localization in the target tissue, and functional improvement following gene transfer therapy.

## MATERIALS AND METHODS

### Animals

All procedures were approved by The Research Institute at the Nationwide Children's Hospital Institutional Animal Care and Use Committee (protocols AR08-00009 and AR06-00054) and performed in accordance with the standards set forth in the eighth edition of Guide for the Care and Use of Laboratory Animals. Wild-type C57BL/6 and mutant C57BL/10ScSn-Dmd*^mdx^*/J (*mdx*) mice were bred and maintained as homozygous animals under standardized conditions [Teklad Global Rodent Diet (3.8% fiber, 18.8% protein, 5% fat chow) and a 12:12 h dark:light cycle] in the Animal Resources Core at The Research Institute at Nationwide Children's Hospital. Although the matched control for the *mdx* mice is wild-type C57BL/10, C57BL/6 mice were used in the present study for the wild-type control group as unpublished data show that the background was unlikely to impact the experimental outcomes assessed in this study.

### rAAVrh74 vector constructs

Four unique vector constructs packaged into the serotype rh74 (rAAVrh74) virus vector were assessed (Fig. 1). Common features of all four constructs include the ABD and CRD of the native dystrophin protein. Two constructs included the ΔR4-23 micro-dystrophin protein, including the H1, H2, and H4 domains, in combination with either an MHCK7 or an MCK promoter region (rAAVrh74.MHCK7.micro-dystrophin and rAAVrh74.MCK.micro-dystrophin, respectively). The rAAVrh74.MHCK7.micro-dystrophin.Cterm construct included the MHCK7 promoter with partial R1 and R24 repeats, H1 and H4 but no H2, and the C-terminal domain. The rAAVrh74.MHCK7.DV.mini-dystrophin construct, which contained the binding site known to anchor nNOS to the membrane (Hakim et al., 2017), used the MHCK7 promotor in combination with a mini-dystrophin ΔR4-15/ ΔR18-R19 gene (resulting from recombination of two vectors: ΔR4-15/ ΔR 18-19/ΔR 24-CT and ΔABD-R19) and included the H1, H3, and H4 hinge domains and the CT. For the rAAVrh74.MHCK7.micro- dystrophin and rAAVrh74.MCK.micro-dystrophin constructs, complementary DNA was codon-optimized for human usage (GenScript, Piscataway, NJ, USA).

The muscle-specific recombinant MCK-based promotors used here have been previously described (Salva et al., 2007). The canonical MCK promoter derived from the muscle creatinine kinase gene (GenBank Accession number M21390)-derived sequence, was used to drive muscle-specific gene expression. The MCK promoter was synthesized by GenScript following derivation from previous work (Shield et al., 1996; Yuasa et al., 2002). It is composed of the mouse MCK enhancer fused to the 351 bp MCK promoter. A 53 bp endogenous mouse MCK exon1 (untranslated) was added downstream of the promoter for efficient transcription initiation (Salva et al., 2007). The recombinant MHCK7 promoter used to drive transgene expression is a muscle-cardiac specific promoter and was based on the MCK promoter and the promoter described by Dr Stephen Hauschka (University of Washington, Seattle, WA, USA) (Salva et al., 2007). This MCK-based promoter used an enhancer derived from the 5′ of the transcription start site within the endogenous muscle CK gene with a proximal promoter (Salva et al., 2007). This enhancer, along with a modified CK7 cassette from the MCK family of genes, was ligated to an α-MyHC enhancer 5′ of the CK portion to enhance cardiac expression (Salva et al., 2007).

The micro-dystrophin constructs included a consensus Kozak sequence, an SV40 intron, and a synthetic polyadenylation site (53 base pairs). The MHCK7 or MCK micro-dystrophin expression cassettes were cloned between AAV2 inverted terminal repeats (ITRs) using flanking *Xba*I restriction enzyme sites. We used the Genewiz proprietary platform to sequence through GC rich regions of the ITR with further confirmation of ITR integrity with restriction enzyme digestion (*Msc*I/*Sma*I).

Each construct was packaged into a rAAVrh74 capsid using the standard triple transfection protocol as previously described (Rodino-Klapac et al., 2007; Schnepp et al., 2005; Sondergaard et al., 2015). A qPCR-based titration method was used to determine an encapsulated vg titer using a Prism 7500 Fast Taqman detector system (PE Applied Biosystems, Waltham, PA, USA) (Schnepp et al., 2005). Vector-specific primer probe sets were used to amplify the sequences directly downstream from the MHCK7 promoter for the three constructs containing MHCK7, and a primer probe for the MCK promoter was used to titer the rAAVrh74.MCK.micro-dystrophin construct as previously described (Pozsgai et al., 2017; Rodino-Klapac et al., 2007). The following primers and probe were used for the MHCK7 and MCK promoters: MHCK7 forward primer, 5′-CCA ACA CCT GCT GCC TCT AAA-3′; MHCK7 reverse primer, 5′-GTC CCC CAC AGC CTT GTT C-3′; MHCK7 probe, 5′-FAM-TGG ATC CCC-Zen-TGC ATG CGA AGA TC-3IABKFQ-3′; MCK forward primer, 5′-CCCGAGATGCCTGGTTATAATT-3′; MCK reverse primer, 5′-GCTCAGGCAGCAGGTGTTG-3′; and MCK probe, 5′-FAMCCAGACATGTGGCTGCTCCCCC-TAMRA-3′ (Pozsgai et al., 2017; Rodino-Klapac et al., 2007).

Constructs were delivered by a single intramuscular injection into the LTA muscle of male *mdx* mice (4-6 weeks of age) at the dose of 1×10^11^ total vg. The contralateral RTA was injected with lactated Ringer's solution, the diluent for the virus, and served as the comparator (*n*=5 *mdx* mice in each group). Mice were necropsied at 4 weeks after vector delivery for assessment of biological and functional endpoints when peak protein expression was reached.

### Immunofluorescence

Shortened dystrophin expression was evaluated by immunofluorescence, as previously described (Pozsgai et al., 2017; Shin et al., 2011). Cryosections (12 µm) from the TA were subjected to immunofluorescent staining for the dystrophin transgene via our previously used protocol (Pozsgai et al., 2017). Sections from the rAAVrh74.MHCK7.micro-dystrophin and rAAVrh74.MCK.micro-dystrophin groups were incubated with a mouse monoclonal human dystrophin primary antibody 4C7 (amino acids 1-68, 1:50; SC-33697, Santa Cruz Biotechnology, Dallas, TX, USA) followed by an Alexa Fluor 568 goat anti-mouse IgG2b secondary antibody (1:250; A21144; Invitrogen, Carlsbad, CA, USA). For the rAAVrh74.MHCK7.micro-dystrophin.Cterm and rAAVrh74.MHCK7.DV.mini-dystrophin groups, sections were incubated with a rabbit polyclonal anti-dystrophin antibody (1:50; ab15277; abcam, Cambridge, MA, USA) followed by an Alexa Fluor 594 goat anti-rabbit secondary antibody (1:250; A32740; Invitrogen).

For percent positive fiber analysis, four random 20X images covering four different quadrants of the muscle section were taken using a Zeiss AxioCam MRC5 camera (San Deigo, CA, USA). Percentage of fibers positive for dystrophin staining (>50% of muscle membrane staining) was determined for each image and averaged for each muscle.

Intensity quantification was determined for the rAAVrh74.MHCK7.micro-dystrophin and rAAVrh74.MCK.micro-dystrophin constructs with BIOQUANT software (version 2019) template (D6) Density Pixel Count for Fluorescence Expression. Other constructs were not evaluated because a different antibody was used for detection. 20x images were measured. To determine the upper and lower threshold for analysis, normal and negative control images were used by selecting the stained membrane, including the various shades of intensity within the run. Threshold settings for the data set were obtained from two trained technical analysts independently. The threshold values obtained for red, green, and blue channels were averaged, with the average value for each channel as the consensus threshold for that data set. A sequence of images was analyzed by selecting the field measurement tool. Values for Fluorescence Quantitation (D6) BIOQUANT measurement algorithm were recorded. An average value was calculated for difference in expression between constructs.

### Morphometric analysis

Central nucleation is an indicator of muscle degeneration/regeneration and is much higher in DMD muscle tissue (Guiraud and Davies, 2019). As a hallmark of dystrophic pathology in the *mdx* mouse, the percentage of myofibers with central nuclei was determined from 12-μm thick cryosections of hematoxylin and eosin-stained TA muscle, as previously described (Rodino-Klapac et al., 2007). Analysis included four fields of 20X magnification per animal per muscle (*n*=4 RTA, *n*=4 LTA, per each treated cohort). Centrally nucleated fibers were quantified using the National Institutes of Health ImageJ software.

### Western blot analysis

Western blot analyses were performed as previously reported (Potter et al., 2021). Protein (50 µg) extracted from LTA and RTA samples from *mdx* mice were separated by sodium dodecyl sulfate–polyacrylamide gel electrophoresis (3-8% Novex NuPAGE gradient gels, Invitrogen, Waltham, MA, USA), blotted on polyvinylidene fluoride membranes and probed with primary monoclonal antibody specific for dystrophin (NCL-DYS3; Leica Biosystems, Richmond, IL, USA) at a dilution of 1:20 followed by Alexa Fluor 680 goat anti-mouse (1:5000, Licor, Lincoln, NE, USA). For the loading control, a GAPDH antibody (Invitrogen) was used at a dilution of 1:2500 followed by a mouse IgG horseradish peroxidase-linked whole antibody derived from sheep (Millipore Sigma, St. Louis, MO, USA) at a dilution of 1:1000.

### Functional assessment

Tetanic contraction of the TA was assessed in intact mice, as previously described (Hakim et al., 2011; Liu et al., 2005; Pozsgai et al., 2016). In brief, 4 weeks following transgene delivery, mice were anesthetized using a ketamine/xylazine mixture, and the TA was secured at patella and distal tendons using double square knots with a 4-0 suture. Mice were then transferred to a thermal-controlled platform and maintained at 37°C. The knee was secured to the metal pin with the patella tendon suture and the distal TA tendon suture to the level arm of the force transducer (Aurora Scientific, Aurora, ON, Canada). An electrode was placed near the sciatic nerve to stimulate it. Once the muscle was stabilized, the resting tension was set to a force (optimal length) where twitch contractions were maximal. After a 3-min rest period, the TA was stimulated at 50, 100, 150, and 200 Hz, allowing a 1-min rest between each stimulus. Absolute force was calculated as follows: force at 150 Hz*9.8 (where 9.8 mN=1 g). TA-specific force was determined as absolute force normalized to cross-sectional area (normalized isometric force or tension: mN/mm^2^). Cross-sectional area was calculated using the following equation: (muscle mass in g)/[(optimal fiber length in cm)×(muscle density in g/cm^3^)], where muscle density is 1.06 g/cm^3^. After functional assessments were completed, the mice were then euthanized, and the TA muscle was dissected out and frozen for histology. An eccentric contraction assay was not performed.

### Digital droplet polymerase chain reaction (*ddPCR*) analysis

ddPCR was performed to quantify the number of vg copies present in targeted muscle, as previously described (Clark et al., 1999; Pozsgai et al., 2016; Rodino-Klapac et al., 2010). Biodistribution analysis was performed on tissue samples collected from vector-dosed *mdx* animals. Tissues were harvested at necropsy, and vector-specific primer probe sets specific for sequences of the MHCK7 or tMCK promoter were used. A vector-specific primer probe set was used to amplify a sequence of the intronic region directly downstream from the promoter that is unique and located within each transgene cassette. To quantify the amount of genomic DNA present in samples, and thus the number of cell nuclei that are present in the sample, a reference gene was used. Copy number was reported as vg copies per nucleus.

### Statistical analysis

Sample sizes of *n*=5 were used to power the study to determine statistical differences in functional evaluation. Additionally, the study was designed and executed as a proof-of-concept evaluation and based on historical gene-therapy publications with similar sample sizes (Zhang et al., 2013; Elkouby et al., 2021). Outlier analyses were run using the ROUT analysis using GraphPad Prism 5 statistical software (Graphpad Software, La Jolla, CA, USA). No outliers were excluded from analysis unless they prematurely died during physiological evaluation. Animals were randomized by body weight prior to start of the study. Personnel who completed data collection, functional evaluation, and analysis were blinded to the treatment group and genotype of animals throughout the study. The correct statistical test for each analysis was determined by first assessing the normality and variance of the data. Normality was evaluated by the Shapiro–Wilks test, as appropriate. Homogeneity of variances was determined by the Levene's test. For parametric data, an one-way analysis of variance (ANOVA) test was used to determine whether there were differences among the group means. Direct comparisons between two groups were evaluated with a student's *t*-test. Data comparisons were considered to be significantly different when *P*<0.05. Data are expressed as the mean±standard error of the mean (s.e.m.; error bars) determined using GraphPad Prism 5.
